# Paramedic intubation

**DOI:** 10.1186/1757-7241-22-S1-A2

**Published:** 2014-07-07

**Authors:** Daniel Davis

**Affiliations:** 1 UCSD Center for Resuscitation Science, California, USA

## 

One of the most controversial topics in resuscitation science is the inclusion of endotracheal intubation (ETI) within the scope of practice for non-physician providers in the prehospital environment. Unfortunately, the medical literature is unable to provide clear evidence for or against this perspective, leading to tremendous variability in practice throughout the world. To better understand this topic, one must address four separate but related issues: 1) the physiological rationale for early intubation during resuscitation, 2) the inconsistency of published reports addressing this controversy, 3) the possibility that suboptimal performance of ETI and subsequence ventilation can explain a lack of outcomes benefit, and 4) the relationship between provider type, training, and airway skills performance. Each of these will be addressed below.

The ABC’s of Resuscitation represent a fundamental paradigm, which recognizes that airway obstruction and hypoxemia often accompany severe injury and illness. Furthermore, addressing these – with ETI representing “definitive” therapy in this regard – may prevent deterioration or reverse secondary injury from hypoxemia. The relationship between hypoxemia and poor outcome from traumatic brain injury (TBI) is well described [1]. Unfortunately, the vast majority of severe TBI patients appear to have pulse oximetry values below 90% [2], underscoring the importance of this issue. In addition, obtunded patients are at risk for aspiration and subsequent pneumonitis or pneumonia, which may further contribute to a critical clinical picture. While these concepts would appear to provide irrefutable support for early intubation in resuscitation, it should be noted that reversing hypoxemia may not prevent hypoxia-mediated injury and that outcomes evidence is conspicuously lacking to support an aggressive approach to airway management.

While the need to control the airway and provide ventilatory support to critically ill and injured patients is rarely challenged, the published literature does not provide definitive evidence in this regard. In fact, early intubation has generally been associated with poor clinical outcomes, with the preponderance of studies performed using non-physician providers leading to the present controversy [3]. Unfortunately, the most likely explanation for these results has less to do with the provider type and more related to research methodology. Despite sophisticated statistical methodologies to adjust for injury severity, the fact remains that patients being considered for invasive airway management are inevitably more critically ill and injured, which almost certainly explains the consistent association between early ETI and increased mortality. More recently, unconventional statistical methodologies and population-based studies have suggested improved outcomes with paramedic ETI, particularly among more severely injured patients [4,5]. In addition, a prospective, controlled trial using advanced practice paramedics in Australia documented improved neurologic outcome with prehospital use of paralytics to facilitate ETI [6].

Although selection bias has clearly influenced the results of prior studies investigating the impact of paramedic ETI on outcome, making it difficult to define an optimal therapeutic approach to airway management during resuscitation, a growing number of studies have identified suboptimal performance of the procedure as well as the subsequent ventilation as critically related to outcome in these patients. The two most important technical components to early ETI that have been identified include avoiding desaturations (particularly with the use of paralytics to facilitate laryngoscopy) and eliminating hyperventilation following successful placement of an advanced airway. Desaturations may exacerbate existing brain injury and can result in asphyxial arrest. Hyperventilation decreases cerebral perfusion via hypocapneic cerebral vasoconstriction (unique to the cerebral vasculature) and via a decrease in cardiac output associated with the rise in intrathoracic pressure that accompanies excessive ventilation rates or prolonged inspiratory times. Both desaturations and hyperventilation have been associated with poor outcomes, and it appears that eliminating these might have reduced or avoided completely the increase in mortality observed during the San Diego Paramedic Rapid Sequence Intubation Trial [7,8]. Both desaturations and hyperventilation can be reduced or eliminated completely with targeted training and equipment interventions. Other opportunities exist to improve clinical outcomes by addressing specific technical issues. These include avoiding the potential dangers of hyperoxemia associated with ETI, minimizing the rise in intracranial pressure during laryngoscopy, and exploring novel strategies for providing ventilation during cardiopulmonary arrest and shock.

The final issue that must be considered with regard to defining the optimal scope of practice for non-physician pre-hospital providers concerns the complex relationships between provider type, procedural experience (both recent and total), training, and available equipment. While these relationships are only just beginning to be elucidated, there appear to be several important observations from the existing literature. First, the type of provider (e.g., physician, nurse, paramedic) appears to be less important than experience and training. Well-trained, experienced flight nurses and paramedics can achieve intubation success and clinical outcomes than rival or even surpass their physician counterparts [9]. Second, it may be difficult for non-physician providers in some pre-hospital systems to accumulate enough clinical experience with certain procedures including ETI to maintain competency. Third, it appears that procedural experience – particularly recent experience – is associated with both performance as well as clinical outcomes [10]. Finally, simulation appears to be able to provide an alternative for clinical experience, although the “exchange rate” for actual clinical encounters remains to be defined [11]. In the future, scope of practice should be related to competency, with better education and training algorithms designed to identify and correct individual deficiencies to assure optimal performance.

In summary, the optimal approach to airway management during resuscitation remains unclear. Although physiological justification exists to support an aggressive approach that includes ETI by non-physician providers, outcomes data remain difficult to interpret and plagued by selection bias. The medical literature appears more clear on the importance of avoiding suboptimal performance of airway management, particularly desaturations and hyperventilation, which can be accomplished via novel approaches to training and equipment. Future efforts must focus on refining technical performance of airway management procedures through better training approaches and equipment as well as elucidating optimal clinical therapeutic interventions.
